# Evaluation of fish biodiversity in estuaries using environmental DNA metabarcoding

**DOI:** 10.1371/journal.pone.0231127

**Published:** 2020-10-06

**Authors:** Hyojin Ahn, Manabu Kume, Yuki Terashima, Feng Ye, Satoshi Kameyama, Masaki Miya, Yoh Yamashita, Akihide Kasai

**Affiliations:** 1 Connectivity of Hills, Humans and Oceans Unit, Kyoto University, Kyoto, Japan; 2 Faculty of Fisheries Sciences, Hokkaido University, Hakodate, Hokkaido, Japan; 3 Center for Environmental Biology and Ecosystem, National Institute for Environmental Studies, Tsukuba, Ibaraki, Japan; 4 Department of Ecology and Environmental Sciences, National History Museum and Institute, Chiba, Japan; University of Hyogo, JAPAN

## Abstract

Biodiversity is an important parameter for the evaluation of the extant environmental conditions. Here, we used environmental DNA (eDNA) metabarcoding to investigate fish biodiversity in five different estuaries in Japan. Water samples for eDNA were collected from river mouths and adjacent coastal areas of two estuaries with high degrees of development (the Tama and Miya Rivers) and three estuaries with relatively low degrees of development (the Aka, Takatsu, and Sendai Rivers). A total of 182 fish species across 67 families were detected. Among them, 11 species occurred in all the rivers studied. Rare fishes including endangered species were successfully detected in rich natural rivers. Biodiversity was the highest in the Sendai River and lowest in the Tama River, reflecting the degree of human development along each river. Even though nutrient concentration was low in both the Aka and Sendai Rivers, the latter exhibited greater diversity, including many tropical or subtropical species, owing to its more southern location. Species composition detected by eDNA varied among rivers, reflecting the distribution and migration of fishes. Our results are in accordance with the ecology of each fish species and environmental conditions of each river.

## Introduction

Threats to biodiversity in aquatic ecosystems have been an issue for decades because of loss of productive habitats [1, 2]. Such environmental perturbations are caused mainly by human influences, through both direct damage to aquatic ecosystems and indirect pollution with sediments, excessive nutrients, and other chemicals. Terrestrial pollutants from agriculture, deforestation, and construction flow into coastal areas through the hydrologic system, mainly through rivers [3–5]. Therefore, humans affect first the estuaries and coastal areas, whose environmental conservation is indicated by the extent of biodiversity. Consequently, comprehensive monitoring of biodiversity is essential for conservation of ecosystems.

Although a number of studies on biodiversity have been reported [6, 7], most of them have focused on local areas of ecologic or economic importance to aquaculture [8], unique ecosystems (e.g., coral reefs, mangroves, tropical islands) [2, 4], and other services [9]. In contrast, biodiversity evaluations that include various regions at the same time have not been carried out, because traditional monitoring methods (observations and/or capture) require considerable financial and labor resources to cover a wide range of habitats [10, 11]. Also, particularly for rare and endangered species, monitoring using traditional methods can negatively affect the organisms and their habitat during the survey.

Here, we tested environmental DNA (eDNA) metabarcoding as a non-invasive and cost-effective method for monitoring the biodiversity of fishes [12] in multiple estuaries at a nation-wide scale. Environmental DNA, defined as genetic material released from organisms into the environment, has become a convenient tool for molecular biology and ecology over the past decade [13, 14]. By sampling soil, sediment, water, and ice, species can be detected even when they cannot be observed visually. This technique was first reported with regard to amphibians [15], followed by fish [16, 17], crustaceans [18], mammals [19], and plants [20]. In addition, combined with next-generation sequencing technology, eDNA enables the processing of massive DNA sequencing data for the identification of various taxa in multiple samples simultaneously, which is termed eDNA metabarcoding [21]. This method is not only practical for assessment of biodiversity, but is also useful to for detection of non-invasive alien, rare, and endangered species while performing a diversity survey [14, 22, 23]. We used universal primers (MiFish-U and MiFish-E) for the metabarcoding process [24].

**Fig 1:**
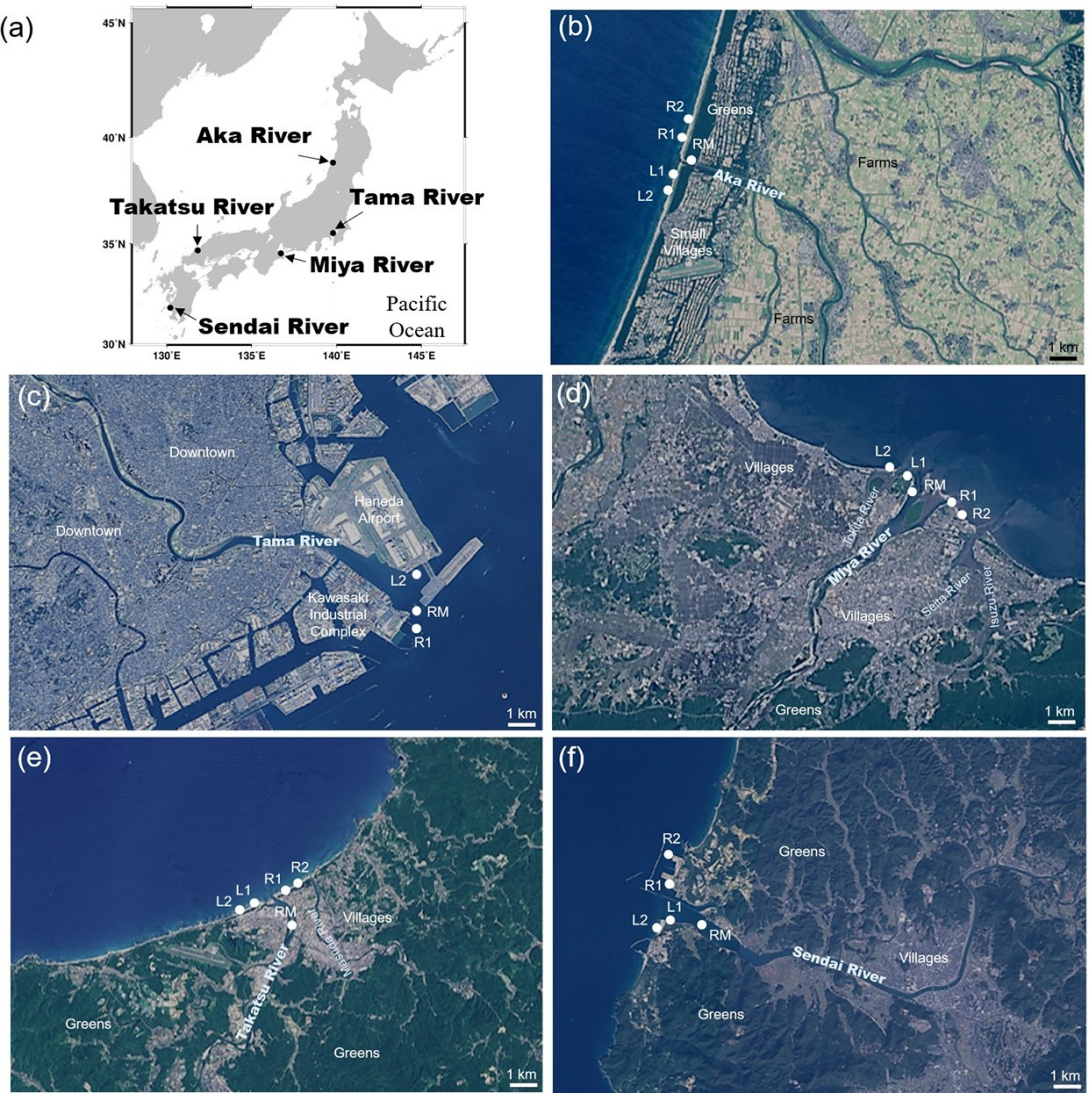
Sampling stations. Location of (a) the five rivers surveyed in this study. Maps showing the location of sampling stations RM (river mouth), L1 (left 500 m), L2 (left 1 km), R1 (right 500 m), and R2 (right 1 km) of (b) Aka River, (c) Tama River, (d) Miya River, (e) Takatsu River, and (f) Sendai River. The satellite photos from (b) to (f) were provided by Geospatial Information Authority of Japan. Scale bar = 1 km.

Five rivers, indicative of different geographical features and human impact on biodiversity, were selected for this study. As Japan stretches extensively from north to south, the latitude of the target rivers varied from 31.85°N to 38.85°N (Fig 1A). The catchment area of the rivers showed considerable variation from natural forest to a megacity. We hypothesized that fish diversity detected from the eDNA survey would reflect those environmental characteristics. Therefore, we confirmed two main aspects. 1) The accordance of fish composition and environment. 2) The relation of biodiversity and human activity.

## Materials and methods

### Water sampling

Five rivers, Aka River (Yamagata prefecture, Tohoku area in northern part; 38.8477 N 139.7863 E at river mouth), Tama River (Tokyo, the capitol city in the middle east; 35.5205 N 139.7978 E at river mouth), Miya River (Mie prefecture, Kansai area in the middle west; 34.5396 N 136.7227 E at river mouth), Takatsu River (Shimane prefecture, Chugoku area in western part; 34.6857 N 131.8291 E at river mouth), and Sendai River (Kagoshima prefecture, Kyushu area in southern part; 31.8417 N 130.2087 E at river mouth) with different geographical features and degrees of urbanization were selected (Fig 1). The water at five stations (at the river mouth, and approximately 500 m and 1 km along the coast on both the left and right sides of the river mouth) was sampled in summer (June or July) 2018. At the river mouth, the water was sampled twice (at high and low tides), and therefore, there was a total of six samples collected from each estuary. For the Tama River, water samples were taken from a boat because the estuary is located between Haneda Airport and the Kawasaki industrial complex, and we could not reach the area from the shore. Moreover, because the airport restricts access to any type of boat near the runway, we could only collect samples from one station of each side of the Tama River estuary and collected four samples instead of six samples (Table 1).

**Table 1:** Environmental conditions of sampling stations.

Aka River (17th, July)	HT	LT	L1	L2	R1	R2
Water temp (°C)	24.1	26	26	26	25.4	25.5
Salinity	6.4	7.7	18.8	29	20.1	29.7
Filtered water (mL)	200	200	600	600	600	600
Tama River (29th, June)	HT	LT	L1	L2	R1	R2
Water temp (°C)	22.3	26.6	-	24.7	23.5	-
Salinity	29.1	22.2	-	24.6	27.9	-
Filtered water (mL)	400	400	-	400	400	-
Miya River (7th, June)	HT	LT	L1	L2	R1	R2
Water temp (°C)	23	22.5	22.2	22.6	23.1	24.7
Salinity	7.45	10.4	25.53	23.39	20.64	21.92
Filtered water (mL)	700	600	200	500	600	600
Takatsu River (16th, July)	HT	LT	L1	L2	R1	R2
Water temp (°C)	26.7	24.1	29	29	27	27
Salinity	0.1	0.1	22.4	23.9	15.1	15.5
Filtered water (mL)	500	600	500	500	500	600
Sendai River (27th, July)	HT	LT	L1	L2	R1	R2
Water temp (°C)	28.7	30	29.7	29.5	29.1	29.3
Salinity	22.2	6.8	14.2	14.6	28.3	29.6
Filtered water (mL)	500	500	500	600	1000	1000

HT: river mouth at high tide; LT: river mouth at low tide; L1: left 500 m; L2: left 1 km; R1: right 500 m; R2: right 1 km.

All sampling and filtering equipment was cleaned with 10% commercial bleach solution. The surface water at each station was sampled by a bucket and immediately filtered using a 0.45-μm polyethersulfone membrane Sterivex filter unit (Merck Millipore, Billerica, MA, USA) and immersed in 1.6 mL RNAlater Stabilization Solution (Thermo Fisher Scientific, Waltham, MA, USA). Water temperature and salinity were measured during sampling. The volume of water samples varied from 200 to 1000 mL depending on turbidity (Table 1). We assumed that variation in sample volume did not affect diversity as we confirmed no correlation between the volume and number of species (r^2^ = 0.0061) by all samples (n = 28). As a negative control, 500 mL of pure water was filtered at each river. Filter units were frozen at -30°C until DNA extraction.

### eDNA extraction

Total DNA was extracted from the Sterivex filter units using a DNeasy Blood and Tissue Kit (Qiagen, Hilden, Germany), following the procedure described by Miya et al. [25] and the manufacturer’s protocol with minor modifications. After removing RNAlater by centrifugation (4,000 × *g* for 2 min), the filter unit was rinsed with sterilized distilled water. For the lysis of eDNA attached to the membrane, proteinase K (20 μL) and lysis buffer AL (200 μL) were applied to the filter unit and incubated inside a 56°C preheated oven for about 20 min. The roller was turned on to enable even collection of DNA from the membrane. After the incubation, the spin column was centrifuged at 4,000 × *g* for 2 min to collect DNA, to which 200 μL of absolute ethanol was then added and mixed well. The resulting solution was transferred to a spin column, centrifuged (6,000 × *g* for 1 min), and then purified twice using wash buffer (AW1 and AW2). After the purification steps, DNA was eluted with the elution buffer (110 μL) provided in the kit. Extracted DNA was stored in a LoBind tube at -30°C.

### Library preparation and sequencing

Samples were sent to the Kazusa DNA Research Institute (Chiba, Japan) for paired-end library preparation and next-generation sequencing (MiSeq) as detailed by Miya et al. [24] and S1 Text. A two-step PCR for paired-end library preparation was employed in the MiSeq platform (Illumina, San Diego, CA, USA). For the first-round PCR (1st PCR), a mixture of the MiFish-U and MiFish-E was used. After completion of the 1st PCR, the purified target products (*ca*. 300 bp) were quantified and diluted. The diluted products were employed as templates for the second-round PCR (2nd PCR) that was carried out with dual-index primers. The blanks were prepared during 1st and 2nd PCR to monitor any contamination. No template was used for both blanks to avoid possible contamination.

### Data preprocessing and taxonomic assignment

Data preprocessing and analysis of MiSeq raw reads were performed with a pipeline (MiFish ver. 2.3) using USEARCH v10.0.240 [26]. The following steps were applied: (1) Forward (R1) and reverse (R2) reads were merged by aligning them with the *fastq_mergepairs* command. (2) Primer sequences were removed from merged reads using the *fastx_truncate* command. (3) Reads without primer sequences underwent quality filtering using the *fastq_filter* command to remove low-quality reads (4) Preprocessed reads were dereplicated using the *fastx_uniques* command and all singletons, doubletons, and tripletons were removed from subsequent analysis as recommended [26]. (5) Dereplicated reads were denoised using the *unoise3* command to generate amplicon sequence variants (ASVs) without any putatively chimeric and erroneous sequences [27]. (6) Finally, ASVs were subjected to taxonomic assignments of species names (molecular operational taxonomic units; MOTUs) using the *usearch_global* command with sequence identity >98.5% to the reference sequences. Above procedures were described in S1 Text in detail.

All negative controls in sampling stations and PCR blanks were also analyzed using this pipeline. The number of reads corresponding to every fish detected in the negative control were deleted (S1 Table) and flathead grey mullet *Mugil cephalus* is removed from Tama River L2 station after this process.

### Species verification

The species obtained by pipeline still needed to be verified because sequencing results comprised only a short region (170 bp) of 12S rRNA [24], and similar sequences might correspond to different species. Also, multiple species could be incorporated into a single species, and *vice versa*. We checked all species on the list with the original aligned sequences using the NCBI Basic Local Alignment Search Tool (http://blast.ncbi.nlm.nih.gov/Blast.cgi), and applied MEGA7 [28] to construct a neighbor-joining tree for all stations characterized by occurrence of the same species. When several species shared the same or similar (>99%) aligned sequence, we confirmed the species identity by referring to species distribution reported by the IUCN (https://www.iucnredlist.org), FishBase (http://www.fishbase.de), illustrated books of Japanese fishes [29–31], and personal communications. For example, the Japanese black porgy (*Acanthopagrus schlegelii*) and the Okinawa seabream (*Acanthopagrus sivicolus*) have the same aligned sequence, but the Okinawa seabream cannot exist in the waters of any station from the present study. On the contrary, we combined two or more species that were considered to be local variations, even if their sequences differed substantially.

Species whose reads number amounted to <0.05% of total reads of library were deleted because they were potentially caused by contamination, as indicated by Andruszkiewicz et al. [32] with some modifications. If species that were obviously not expected in this area were detected, but represented commonly consumed food items (e.g. Allaska pollock, Tuna), they were regarded as contamination and removed as well.

### Estimates of biodiversity

Even if fish biomass could be reportedly determined by eDNA [33], eDNA has been limited to certain species. Moreover, it has not been applied to metabarcoding because of species-specific amplification rates [34], environment-dependent degradation rates [16, 35], and PCR inhibition by environmental factors [10, 12]. Therefore, the estimate of biomass requires a complex model and the possible use of eDNA for this purpose needs to be verified. Biodiversity is sometimes calculated by functions such as ‘number of species’ and ‘biomass;’ however, as biomass information was not available in the present study, we considered ‘species richness’ as a proxy for ‘biodiversity.’

### Environmental data set

Data regarding nutrients were obtained from the Ministry of the Environment of Japan (http://water-repo.env.go.jp/water-repo/). We used the annual mean value of nutrient concentration combining total nitrogen (TN) and total phosphorus (TP) published in the Measurement Results of Water Quality in Public Waters in FY 2016 (Ministry of the Environment) as a water quality index of the river. The annual mean value is based on 6–12 measurements a year at each monitoring point. The monitoring points corresponding to the target watersheds (points using the TN and TP values) were the most downward points of each river.

The revetment rate was calculated by measuring the distance of artificially protected areas, such as concrete-sealed piers or concrete tetrapods, within a distance of 3 km on both sides of the river and shore from the river mouth, using Google Earth Pro (http://support.google.com/earth/answer/21995?hl=ja).

### Statistical analysis

To examine the effect of salinity or water temperature on the ratio of freshwater, brackish, or seawater species, we used general linear models (GLMs) with a negative binominal distribution and a log link function. To this end, we applied the *glm*.*nb* function in the *MASS* package. The number of freshwater, brackish, or seawater species (classified by Nelson [36]) in each sample was used as a response variable; salinity or water temperature were explanatory variables; and the total number of fish species represented an offset term. To verify the accuracy of the six models, the areas under the Receiver Operating Characteristic curves (AUCs) were calculated, using the *roc* function in the *pROC* package [37]. Accuracy was defined as low (AUC < 0.7), moderate (0.7 ≤ AUC < 0.9), and high (AUC ≥ 0.9) (Table 2).

**Table 2:** Summary of models^†^ used to assess the effect of each environmental factor on the rate of freshwater, brackish, or marine fish.

Variable	Freshwater species	Brackish water species	Seawater species
**Effect of salinity**			
(Intercept)	2.615***	2.884***	1.688***
Salinity	-0.046**	-0.008	0.042***
AUC	0.839	0.825	0.838
**Effect of water temperature**			
(Intercept)	4.377**	2.114***	-1.525*
Water temperature	-0.098	0.024	0.152***
AUC	0.856	0.841	0.961

^†^Based on comparison of null and full models in general linear model results; β coefficients of predictor variables are shown.

Abbreviations: AUC, area under the Receiver Operating Characteristic curve

*p < 0.05

** *p* < 0.01, and

***p < 0.001 in a Ward test

To examine the human impact on the number of fish species, we again applied the above GLMs using the *glm*.*nb* function in the *MASS* package. The number of species in each river was used as a response variable. We used data about TN, TP, and revetment rates as indicators of human impact. However, both TN and TP had a high variance inflation factor (VIF), which indicated high multicollinearity among these variables (VIF = 26.1 and 15.6 for TN and TP, respectively, VIF = 7.3 for revetment rate). After removal of TP, there was no multicollinearity between TN and revetment rate (VIF = 7.0), so we used TN and revetment rates as explanatory variables for our GLM analyses. These VIF values were calculated using the *vif* function in the *car* package [38]. The number of samples was used as an offset variable. For model selection among GLMs, we used the *dredge* function in the *MuMIn* package [39]. The best model was selected using Akaike’s information criterion (AIC), which stipulates that the best model for any candidate set applied to a given data set is that with the lowest AIC value. Following Burnham and Anderson [40], models with ΔAIC < 2 were assumed to be reasonable alternatives to the best model and thus were retained (Table 3).

**Table 3:** Summary of models with ΔAIC < 2^†^ used to assess the effect of human impact on the number of fish species.

Model	Variable			Weight	df	AIC	ΔAIC
	(Intercept)	TN	Revetment				
1	4.557***	-0.214***		0.608	3	76.02	0
2	4.552***	-0.24	0.001	0.231	4	77.96	1.94

^†^Based on comparison of null and full models in general linear model results; β coefficients of predictor variables are shown.

Abbreviations: AIC, Akaike’s information criterion; TN: total nitrogen

***p < 0.001 in a Ward test

All statistical tests were carried out using R software ver. 3.5.2 [41].

## Results

### Species occurrence

**Fig 2:**
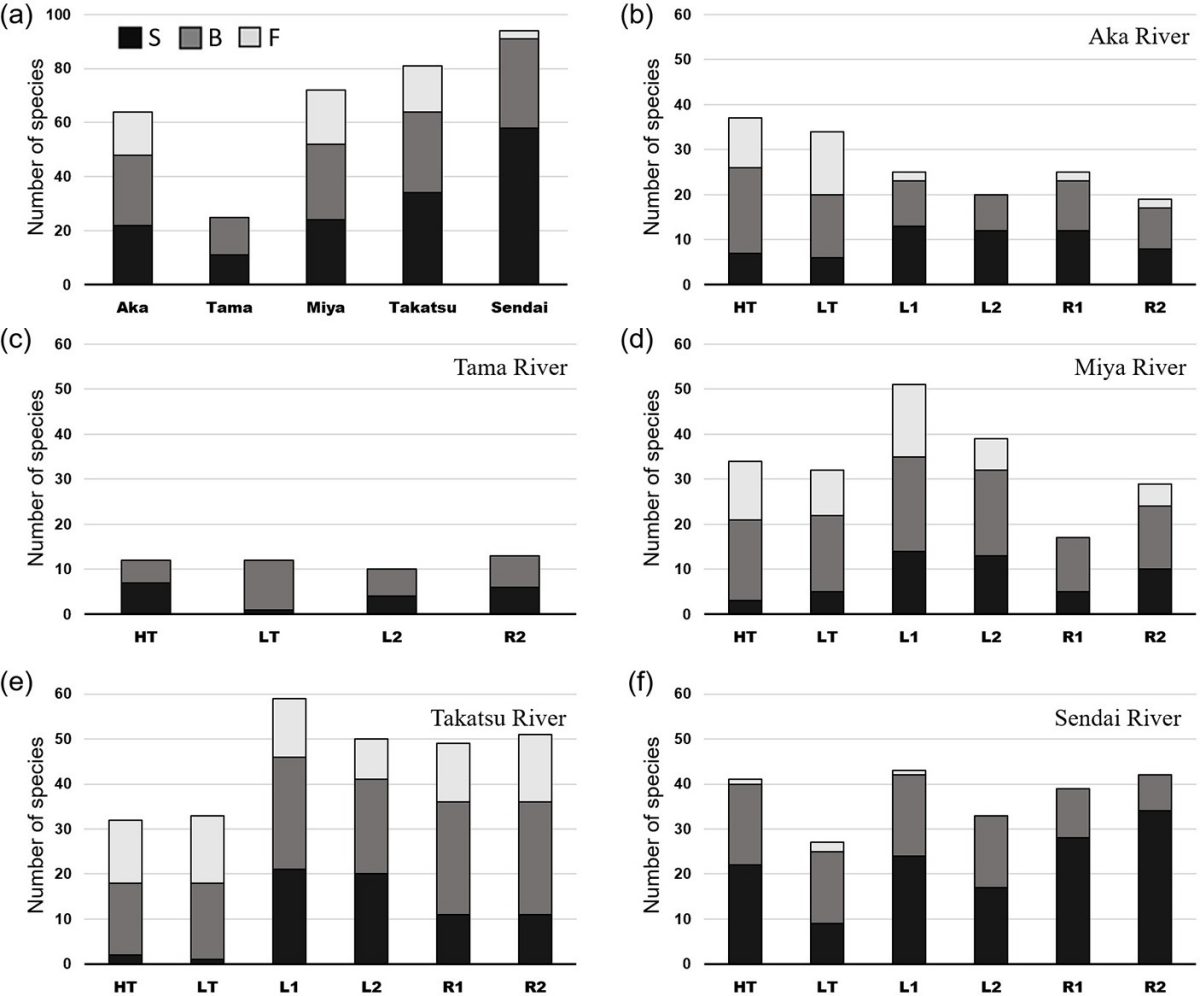
Species richness. Number of species present in (a) all five rivers and at each station, (b) Aka River, (c) Tama River, (d) Miya River, (e) Takatsu River, and (f) Sendai River. HT: river mouth at high tide; LT: river mouth at low tide; L1: left 500 m; L2: left 1 km; R1: right 500 m; R2: right 1 km; S: seawater species; B: brackish water species having a wide range of salinity tolerance including migrating fishes; F: freshwater species.

A total of 182 species from 67 families were detected in the present eDNA survey (S2 Table). Most species (94) occurred in the Sendai River and fewest (25) in the Tama River; whereas the Aka, Miya, and Takatsu Rivers contributed with 64, 72, and 81 species, respectively (Fig 2A). Eleven species commonly observed in Japanese coastal areas (yellowfin goby *Acanthogobius flavimanus*, blackhead seabream *Acanthopagrus schlegelii*, common carp *Cyprinus carpio*, Japanese anchovy *Engraulis japonicus*, largescale blackfish *Girella punctata*, dotted gizzard shad *Konosirus punctatus*, Japanese sea bass *Lateolabrax japonicus*, flathead grey mullet *Mugil cephalus*, *Parablennius yatabei*, *Platycephalus* sp. 2, and *Takifugu* spp.) were reported in all five estuaries.

*Cobitis takatsuensis*, red stingray *Hemitrygon akajei*, Japanese jack mackerel *Trachurus japonicus* (NT), common carp, Japanese seahorse *Hippocampus mohnikei* (VU), Japanese eel *Anguilla japonica*, and redspotted grouper *Epinephelus akaara* (EN) are endangered according to the IUCN red list (https://www.iucnredlist.org). Moreover, *C*. *takatsuensis* and Japanese eel are registered as endangered species at the EN level by the Ministry of the Environment of Japan (www.env.go.jp). An additional 11 species, detected by eDNA, including *Eutaeniichthys gilli*, *Gymnogobius castaneus*, oriental weather loach *Misgurnus anguillicaudatus*, cherry salmon, *Sarcocheilichthys variegatus*, slender bitterling *Tanakia lanceolata* (NT), fourspine sculpin *Cottus kazika*, *Cottus reinii*, dark sleeper *Odontobutis hikimius* (VU), Japanese fluvial sculpin *Cottus pollux*, and *Gymnogobius scrobiculatus* (EN), are considered as endangered in Japan (http://ikilog.biodic.go.jp/Rdb/env).

### Habitat composition of each river

A detailed station-by-station analysis (Fig 2B–2F) revealed that in the Tama River, freshwater species were not detected from all stations at the estuary (Fig 2C; S2 Table). Only a small proportion of freshwater species occurred at the river mouth and at the station 500 m left along the coast from the mouth of the Sendai River, while no freshwater species occurred at the other stations (Fig 2F). In the Aka River, freshwater species accounted for 30–40% of total species at the river mouth, but decreased quickly to fewer than 10% along both the left and right sides of the coast. In contrast, seawater species increased at stations in the coastal area (Fig 2B). Similar results were obtained for the Takatsu River, with the proportion of freshwater species decreasing and that of seawater species highly increasing in the coastal area (Fig 2E). In the Miya River, freshwater species decreased in the coastal area, except for the station at 500 m on the left side (Fig 2D).

A different result was observed regarding the number of species in the Aka and Takatsu Rivers (Fig 2B and 2E). More species were detected at the river mouth (37 species at high tide and 34 species at low tide) of the Aka River than in its surrounding coastal area (19–25 species). In the Takatsu River, diversity was higher in the coastal area (49–59 species) than at the river mouth (32 at high tide and 33 species at low tide). The number of species in the Sendai River decreased during low tide (27 species) compared to high tide (41 species) at the river mouth (Fig 2F). In the Tama River, species composition changed at the river mouth as the tide switched from high to low and seawater species decreased on the low tide, even though the total number of species (12 species) remained the same (Fig 2C). No distinguishable change was found between high and low tides at the river mouth of the other three rivers.

The best models examining the effect of salinity or water temperature on the ratio of freshwater, brackish, or seawater species could be obtained with relatively high accuracy (AUC = 0.825–0.961; Table 2). The proportion of freshwater species decreased as salinity increased (*p* < 0.01), whereas that of seawater species increased as salinity increased (*p* < 0.001) for all five rivers. In contrast, the proportion of brackish water fish was not affected by salinity. On the one hand, the proportion of seawater species increased at higher water temperatures (*p* < 0.001), while water temperature had no significant effect on brackish and freshwater species (*p* > 0.05).

### Relationships between environmental factors and the number of species

**Fig 3:**
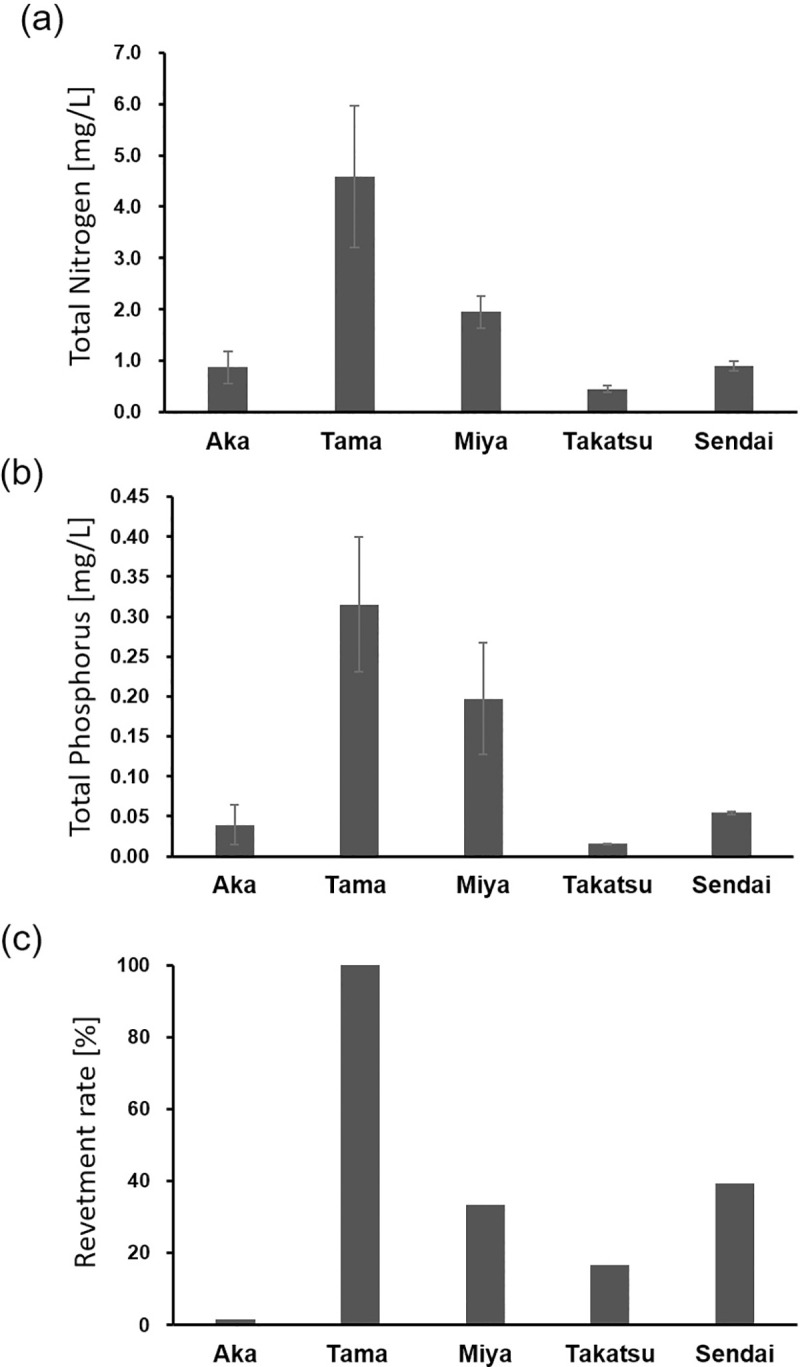
Human effects. Nutrients (mean ± SD) including (a) total nitrogen [mg/L] and (b) total phosphorus [mg/L] of the five rivers based on 2016 data obtained from the Ministry of the Environment, Japan (https://water-repo.env.go.jp/water-repo/). (c) Revetment rate [%] of the five rivers calculated using Google Earth Pro (2018 Google Image Landsat/Copernicus, US Dept of State Geographer Data SIO, NOAA, U. S. Navy, NGA, GEBCO). Bars show standard deviations.

Nutrient concentration (TN and TP) was highest in the Tama River (Fig 3), which flows through a mega city (Fig 1), and relatively low in the Aka and Takatsu Rivers, which flow through rural areas. A similar result was obtained regarding the revetment rate.

Among the GLMs for evaluating the effect of human impact on the number of fish species, two models with ΔAIC < 2 were retained (Table 3). Both models included TN, whereby the number of species increased as TN decreased (*p* < 0.001). In the 2nd model, revetment was included but it had no significant effect (*p* > 0.05).

## Discussion

### Distribution of detected species

The 11 species detected in all five rivers are common in Japan, and some of them (e.g., common carp and flathead grey mullet) have a worldwide distribution [42, 43]. Some, such as yellowfin goby and *Takifugu* spp., can tolerate various environmental conditions [44, 45]. On the contrary, the endemic species *C*. *takatsuensis* was found only in a single habitat (i.e., the Takatsu River; S2 Table), confirming its known limited distribution [46]. This species is registered as an endangered species on the IUCN red list together with seven other species found in this study (https://www.iucnredlist.org). It is of particular importance that the endangered species were successfully detected by the eDNA survey as it is a non-intrusive method for both the environment and the subjects [12]. Therefore, eDNA could be applied not only for biodiversity research, but also to detect rare, endangered species [33]. Also, tropical to subtropical species (e.g., *Caranx ignobilis* [47]; *Spratelloides gracilis* [48]), only occurred in the Sendai River (S2 Table), which is located at the southernmost sampling station of the study. These results were in accordance with the distribution of fish species.

### Environmental conditions and biodiversity

Biodiversity is closely related to the environmental conditions [8]. The results of GLMs showed that salinity affected the proportion of freshwater and seawater fishes, which varied among the five rivers. Specifically, no freshwater species eDNA samples were detected in the Tama River, which can be explained by the sampling stations being near the coast and salinity being over 20 (Table 1; Fig 1C). The proportion of seawater species accounted for more than 50% at high tide but decreased notably at low tide (Fig 2C). The Sendai River showed a very small proportion of freshwater species at the river mouth, which is relatively wide (>1 km), compared with Aka, Miya and Takatsu River (Figs 1 and 2F). It is believed that seawater easily enters into rivers with wide mouths, which causes freshwater from the river to disperse and dilute across the adjacent coastal areas. As a result, brackish and seawater species accounted for more than 90% of hits in this case.

Besides the width of rivers, tidal range is another factor with a strong influence on species composition. The tidal ranges are very small in the Sea of Japan [49], ranging from 6 cm for the Aka River to 55 cm for the Takatsu River, on the day of the sampling (www.jma.go.jp). In contrast, the tidal range of the Tama, Miya, and Sendai Rivers, which are located on the Pacific coast, was 167 cm, 67 cm, and 227 cm, respectively. Not surprisingly, salinity and number of species differed between high and low tides in the Sendai River (Table 1; Fig 2F). In the Tama River, the number of species did not differ between high and low tides; however, seawater species decreased at low tide (Fig 2C).

Species composition in the Aka River differed remarkably between the river mouth and coastal area; the proportion of freshwater species was about 30–40% at the river mouth but decreased to 8–10% in the coastal area, whereas seawater species increased from 18–19% at the river mouth to 42–60% in the coastal area. This pattern can also be explained by the width of the river mouth, which is very narrow (*ca*. 100 m) and thus affects species composition (Figs 1B and 2B). A similar trend was observed for the Takatsu River, which also has a narrow river mouth (<300 m); freshwater species decreased and seawater species increased in the coastal area. The proportion of seawater species was especially small at the river mouth of the Takatsu River, where water sampled from the bridge located about 1 km away from the river mouth had a salinity of 0.1 at both high and low tides (Table 1; Figs 1E and 2E). In fact, GLM analysis revealed that salinity had a significant effect on the proportion of freshwater and seawater species (Table 2).

Biodiversity was high at the river mouth of the Aka River, and in the coastal area of the Takatsu River (Fig 2B and 2E; S2 Table). As the number of species was almost identical at the river mouth of both rivers (34–37 species and 32–33 species, respectively), the observed change in biodiversity could be explained by two phenomena. First, as mentioned above, there are fewer freshwater species in the coastal area of the Aka River. Second, marine biodiversity is higher in the Takatsu River because it is located in the southern part of Japan and in general biodiversity increases toward lower latitudes [50]. GLM results supported the increase in number of seawater species when water temperature increased (Table 2).

Composition and number of species were less straightforward for the Miya River, reflecting its complex geography and environment (Fig 1D). For example, the number of species was highest at the station 500 m along the left of the river mouth (Fig 2D), which can be explained by the junction of two rivers, the Miya River and the Tokita River. However, the number of species was lowest at the station 500 m to the right of the river mouth, where no freshwater species were detected; the reason for this was not clear. The narrow river mouth beside the sampling station (R1) might prevent the flow of freshwater to the right side of the coast, but salinity was lower on the right side than on the left side, and some freshwater species were detected at the station 1 km to the right. One of the limitations and weaknesses of eDNA is the low amount of extracted DNA, which may not be enough for amplification and comprehensive species detection, as well as the presence of inhibitors such as humic acid, which might affect the results [14]. Therefore, although generally accurate, eDNA results might not always reflect all species present and other factors should be considered [51, 52].

On the other hand, it is another interesting founding of present study that freshwater species were detected from adjacent coastal area by eDNA where freshwater species actually cannot exist. It can be referred that we collected eDNA moved by waterflow and the pattern differed by river (Fig 2). It would be worth to investigate about how far eDNA from river can transport to ocean and its relation with flow system. This could give a clue for eDNA localization in future study.

### Effect of human activity

Human activity exerts a large influence on the environment and biodiversity [5, 6]. Water quality is closely related to the biodiversity of aquatic animals [9]. Using nutrient concentrations (TN and TP) and revetment rate as indices of human activity and urbanization, we determined the impact of humans on biodiversity. GLM results indicated that TN significantly affected biodiversity, whereas the revetment rate had no effect (Table 3). The Tama River, which had the lowest biodiversity (Fig 2A), had the highest values for TN, TP, and revetment rates (Fig 3). The degree of urbanization of the Tama and Miya Rivers can be inferred not only from the concentration of nutrients and revetment rate but also from satellite images (Fig 1C and 1D). Even though the shoreline of the Sendai River has been extensively modified for flood control so that its revetment rate is now as high as for the Miya River, the surrounding area of the Sendai River has remained untouched and the nutrient concentration remains low (Figs 1 and 3). The Miya River showed relatively high biodiversity because of its location in the southern part of Japan along the Pacific coast, which is affected by the Kuroshio warm current. In comparison, even though it is located in the northern part of Japan, biodiversity was quite high in the Aka River (Fig 2A), which can be explained by the vastly pristine environment of the river (Fig 1B). This is an important result as it indicates that efforts to conserve the environment can also improve biodiversity. Both the Takatsu and Sendai Rivers showed high biodiversity with low human effect and geographical location (Figs 1 to 3).

## Conclusion

The present study demonstrates that eDNA is a convenient tool for monitoring the distribution, migration, and diversity of fishes. By simply collecting 1 L of water, we successfully detected 182 species including endangered species, covering a wide range of areas in a short period. Even though our experimental design has limitation that it is just a case study with single day sample, the number and list of species from obtain results reflected the ecology of each fish and environmental conditions, such as eutrophication and temperature, in each river. We believe further development of the eDNA technique will offer an alternative method for accurate and non-invasive monitoring of aquatic life.

## Supporting information

S1 Text Entire procedure of library preparation, sequencing, data preprocessing and taxonomic assignment. (PDF)

S1 Table Summary of data preprocessing steps and subsequent taxon assignment using pipeline analysis (MiFish ver. 2.3). Numerals are read numbers and those in parentheses of “Data preprocessing” are percentages against the raw read numbers, while those in “Taxon assignment” are percentages against the denoised read numbers.
(PDF)

S2 Table List of species detected at the sampling stations. Plus (+) represents occurrence. HT: river mouth at high tide; LT: river mouth at low tide; L1: left 500 m; L2: left 1 km; R1: right 500 m; R2: right 1 km. ^†^: endangered species according to the IUCN (https://www.iucnredlist.org). ^‡^: endangered species according to the Ministry of the Environment of Japan (http://ikilog.biodic.go.jp/Rdb/env). ^†‡^: endangered species according to both classifications.
(PDF)
