# Supplementary material for: Evaluation of fish biodiversity in estuaries using environmental DNA metabarcoding
Source: PLoS One. 2020 Oct 6;15(10):e0231127. doi: 10.1371/journal.pone.0231127 (PMC7538199; doi:10.1371/journal.pone.0231127)
Supplement: S1 Text — (PDF) [file pone.0231127.s001.pdf]

Text S1

Library preparation and sequencing

A two-step PCR for paired-end library preparation was employed in the MiSeq platform (Illumina, San Diego, CA, USA). For the first-round PCR (1st PCR), a mixture of the following four primers was used: MiFish-U-forward (5′–ACA CTC TTT CCC TAC ACG CTC TTC CGA TCT NNN GTC GGT AAA ACT CGT GCC AGC–3′), MiFish-U-reverse (5′–GTG ACT GGA GTT CAG ACG TGT GCT CTT CCG ATC TNN NNN NCA TAG TGG GGT ATC TAA TCC CAG TTT G–3′), MiFish-E-forward-v2 (5′–ACA CTC TTT CCC TAC ACG CTC TTC CGA TCT NNN RGT TGG TAA ATC TCG TGC CAG C–3′), and MiFish-E-reverse-v2 (5′–GTG ACT GGA GTT CAG ACG TGT GCT CTT CCG ATC TNN NNN NGC ATA GTG GGG TAT CTA ATC CTA GTT TG–3′). These primer pairs amplified a hypervariable region of the mitochondrial 12S rRNA gene (*ca*. 172 bp; hereafter called "MiFish sequence") and appended primer-binding sites (5′ ends of the sequences before six Ns) for sequencing at both ends of the amplicon. The six random bases (Ns) were used in the middle of these primers to enhance cluster separation in the flow cells during initial base call calibrations of the MiSeq platform.

The 1st PCR was carried out with 35 cycles of a 12-μL reaction volume containing 6.0 μL 2 × KAPA HiFi HotStart ReadyMix (KAPA Biosystems, Wilmington, MA, USA), 2.8 μL of a mixture of the four MiFish primers in equal volumes (U/E forward and reverse primers; 5 μM), 1.2 μL sterile distilled water, and 2.0 μL eDNA

template (a mixture of the duplicated eDNA extracts in equal volumes). To minimize PCR dropouts during the 1st PCR, eight replications were performed for the same eDNA template using a strip of eight tubes (0.2 μL). After an initial 3 min denaturation at 95°C, the thermal cycle profile (35 cycles) was as follows: denaturation at 98°C for 20 s, annealing at 65°C for 15 s, and extension at 72°C for 15 s. There was a final extension at 72°C for 5 min. The 1st PCR blanks were prepared during this process in addition to negative controls for each river.

After completion of the 1st PCR, equal volumes of the PCR products from the eight replications were pooled in a single 1.5-mL tube and purified using a GeneRead Size Selection kit (Qiagen) following the manufacturer’s protocol for the GeneRead DNA Library Prep I Kit. Accordingly, column purification was performed twice to completely remove adapter dimers and monomers. Subsequently, the purified target products (*ca*. 300 bp) were quantified using TapeStation D1000 (Agilent Technologies, Tokyo, Japan), after diluting them to 0.1 ng μL^{-1} with Milli Q water. The diluted products were employed as templates for the second-round PCR (2nd PCR).

For the 2nd PCR, the following two primers were used to append dual-index sequences (eight nucleotides indicated by Xs) and flow cell-binding sites for the MiSeq platform (5′ ends of the sequences before eight Xs): 2nd-PCR-forward (5′–AAT GAT ACG GCG ACC ACC GAG ATC TAC ACX XXX XXX XAC ACT CTT TCC CTA CAC GAC GCT CTT CCG ATC T–3′) and 2nd-PCR-reverse (5′–CAA GCA GAA GAC GGC ATA CGA GAT XXX XXX XXG TGA CTG GAG TTC AGA CGT GTG CTC TTC CGA TCT–3′).

The 2nd PCR was carried out with 10 cycles in a 15-μL reaction volume containing 7.5 μL 2 × KAPA HiFi HotStart ReadyMix, 0.9 μL of each primer (5 μM), 3.9 μL sterile distilled water, and 1.9 μL template (0.1 ng μL^{-1} except for the three blanks). After an initial 3 min denaturation at 95°C, the thermal cycle profile (10 cycles) was as follows: denaturation at 98°C for 20 s, combined annealing and extension at 72°C for 15 s. There was a final extension at 72°C for 5 min. The blank for the 2nd PCR was prepared during this process as well as to monitor any contamination. No template was used for the 1st and 2nd PCR to avoid possible contamination.

All dual-indexed libraries were pooled in equal volumes into a 1.5-mL tube. Then, the pooled dual-indexed library was separated on a 2% E-Gel Size Select agarose gel (Life Technologies, Carlsbad, CA, USA) and the target amplicons (*ca.* 370 bp) were retrieved from the recovery wells using a micropipette. The concentration of the size-selected libraries was measured using a Qubit dsDNA HS assay kit and a Qubit fluorometer (Life Technologies). The libraries were diluted to 12.0 pM with HT1 buffer (Illumina) and sequenced on the MiSeq platform using a MiSeq v2 Reagent Kit for 2 × 150 bp PE (Illumina) following the manufacturer's protocol.

Data preprocessing and taxonomic assignment

Data preprocessing and analysis of MiSeq raw reads were performed with a pipeline (MiFish ver. 2.3) using USEARCH v10.0.240 [1]. The following steps (summarized in S1 Table) were applied: (1) Forward (R1) and reverse (R2) reads were merged by aligning them with the *fastq_mergepairs* command. During this process, the following

reads were discarded: low-quality tail reads with a cut-off threshold set at a quality (Phred) score of 2, reads that were too short (<100 bp) after tail trimming, and paired reads with multiple differences (>5 positions) in the aligned region (*ca*. 65 bp). (2) Primer sequences were removed from merged reads using the *fastx_truncate* command. (3) Reads without primer sequences underwent quality filtering using the *fastq_filter* command to remove low-quality reads with an expected error rate >1% and reads that were too short (<120 bp). (4) Preprocessed reads were dereplicated using the *fastx_uniques* command and all singletons, doubletons, and tripletons were removed from subsequent analysis as recommended [1]. (5) Dereplicated reads were denoised using the *unoise3* command to generate amplicon sequence variants (ASVs) without any putatively chimeric and erroneous sequences [2]. (6) Finally, ASVs were subjected to taxonomic assignments of species names (molecular operational taxonomic units; MOTUs) using the *usearch_global* command with sequence identity >98.5% to the reference sequences. ASVs with sequence identities of 80–98.5% were tentatively assigned "U98.5" labels before the corresponding species name with the highest identity (*e.g.*, U98.5_*Pagrus_major*) and they were subjected to clustering at the 0.985 level using the *cluster_smallmem* command. In an incomplete reference database, this clustering step enables the detection of multiple MOTUs under an identical species name. We annotated such multiple MOTUs with "gotu1, 2, 3…" and tabulated all of these outputs (MOTUs plus U98.5_MOTUs) with read abundances. We excluded ASVs with sequence identities <80% (saved as "no_hit") from the above taxonomic assignments and downstream analyses because all of them were found to be non-fish

organisms.

As a reference database, we assembled MiFish sequences from Masaki Miya’s laboratory. In addition, we downloaded all fish whole mitochondrial genome and 12S rRNA gene sequences from NCBI as of 26 June 2017 and extracted MiFish sequences using a custom Perl script [3]. We combined the MiFish sequences from the two sources in a FASTA format and used the combined sequences as the custom reference database for taxonomic assignments. The final reference database consisted of 27,871 sequences from 7,555 species belonging to 2,612 genera and 464 families.

We refined the above automatic taxonomic assignments with reference to family-level phylogenies based on MiFish sequences from MOTUs, U98.5_MOTUs, and the reference sequences from those families. For each family, we assembled representative sequences (most abundant reads) from MOTUs and U98.5_MOTUs, and added all reference sequences from that family and an outgroup (a single sequence from a closely-related family) in a FASTA format. We subjected the FASTA file to multiple alignment using MAFFT [4] with a default set of parameters. We constructed a neighbor-joining tree with the aligned sequences in MEGA7 [5] using Kimura two-parameter distances. The distances were calculated using pairwise deletion of gaps and among-site rate variations modeled with gamma distributions (shape parameter = 1). We performed bootstrap resamplings (n = 100) to estimate statistical support for internal branches of the neighbor-joining tree and to root the tree with the outgroup, in order to reduce false sequence from the reference database.

We inspected a total of 82 family-level trees and revised the taxonomic

assignments. For U98.5_MOTUs placed within a monophyletic group consisting of a single genus, we assigned that genus to unidentified MOTUs with “sp” plus sequential numbers (e.g., *Pagrus* sp1, sp2, sp3, ...). For the remaining MOTUs ambiguously placed in the family-level tree, we assigned the family name with “sp” plus sequential numbers (e.g., Sparidae sp1, sp2, sp3, ...).

All negative controls in sampling stations and PCR blanks were also analyzed using this pipeline. The number of reads corresponding to every fish detected in the negative control were deleted (S1 Table) and flathead grey mullet *Mugil cephalus* is removed from Tama River L2 station after this process.

References

1. Edgar RC. Search and clustering orders of magnitude faster than BLAST. Bioinformatics. 2010; 26: 2460–2461.
2. Callahan BJ, McMurdie PJ. Holmes SP. Exact sequence variants should replace operational taxonomic units in marker-gene data analysis. ISME J. 2017; 11: 2639–2643.
3. Miya M, Sado T. Multiple species detection using MiFish primers. In: eDNA Methods Standardization Committee editors. Environmental DNA sampling and

experimental manual ver. 2.1. The eDNA Society, Otsu, Japan; 2019. pp. 55-92.

4. Katoh K, Toh H. Recent developments in the MAFFT multiple sequence alignment program. Brief Bioinformatics. 2008; 9: 286–298.
5. Kumar S, Stecher G, Tamura K. MEGA7: molecular evolutionary genetics analysis version 7.0 for bigger datasets. Mol Biol Evol. 2016; 33: 1870–1874.
