# Supplementary material for: Evaluation of fish biodiversity in estuaries using environmental DNA metabarcoding
Source: PLoS One. 2020 Oct 6;15(10):e0231127. doi: 10.1371/journal.pone.0231127 (PMC7538199; doi:10.1371/journal.pone.0231127)
Supplement: S1 Table — Numerals are read numbers and those in parentheses of “Data preprocessing” are percentages against the raw read numbers, while those in “Taxon assignment” are percentages against the denoised read numbers. (PDF) [file pone.0231127.s002.pdf]

S1 Table.

Library	Raw read	Data preprocessing			Taxon assignment	
		Merged	Quality filtered	Denoised	Fish	Non-fish
Aka (High tide)	118,788	115,802 (97.5)	114,784 (96.6)	103,907 (87.5)	103,835 (99.9)	72 (0.1)
Aka (Low tide)	103,740	101,039 (97.4)	100,086 (96.5)	90,209 (87.0)	90,199 (100)	10 (0.0)
Aka (Left 500 m)	194,918	190,839 (97.9)	189,593 (97.3)	180,918 (92.8)	180,914 (100)	4 (0.0)
Aka (Left 1 km)	173,568	166,071 (95.7)	164,763 (94.9)	153,667 (88.5)	153,627 (100)	40 (0.0)
Aka (Right 500 m)	123,351	120,362 (97.6)	119,344 (96.8)	111,007 (90.0)	110,988 (100)	19 (0.0)
Aka (Right 1 km)	128,843	125,802 (97.6)	124,983 (97.0)	118,721 (92.1)	118,721 (100)	0 (0.0)
Tama (High tide)	79,490	71,151 (89.5)	70,543 (88.7)	65,652 (82.6)	65,627 (100)	25 (0.0)
Tama (Low tide)	248,331	172,848 (69.6)	170,955 (68.8)	159,290 (64.1)	159,234 (100)	56 (0.0)
Tama (Left 1 km)	284,947	164,667 (57.8)	162,990 (57.2)	151,642 (53.2)	150,851 (99.5)	791 (0.5)
Tama (Right 500 m)	258,258	201,511 (78.0)	199,299 (77.2)	186,924 (72.4)	186,742 (99.9)	182 (0.1)
Miya (High tide)	113,939	111,758 (98.1)	110,988 (97.4)	102,246 (89.7)	102,242 (100)	4 (0.0)
Miya (Low tide)	95,202	93,526 (98.2)	92,919 (97.6)	86,238 (90.6)	86,233 (100)	5 (0.0)
Miya (Left 500 m)	125,026	120,839 (96.7)	119,879 (95.9)	108,768 (87.0)	107,356 (98.7)	1,412 (1.3)
Miya (Left 1 km)	83,342	81,639 (98.0)	81,105 (97.3)	74,467 (89.4)	74,355 (99.8)	112 (0.2)
Miya (Right 500 m)	73,975	72,479 (98.0)	71,979 (97.3)	66,688 (90.2)	66,688 (100)	0 (0.0)
Miya (Right 1 km)	98,725	96,859 (98.1)	96,148 (97.4)	89,019 (90.2)	88,991 (100)	28 (0.0)
Takatsu (High tide)	168,953	164,080 (97.1)	162,519 (96.2)	149,184 (88.3)	148,611 (99.6)	573 (0.4)
Takatsu (Low tide)	214,832	209,021 (97.3)	206,950 (96.3)	189,960 (88.4)	188,096 (99.0)	1,864 (1.0)
Takatsu (Left 500 m)	194,703	186,526 (95.8)	185,168 (95.1)	169,508 (87.1)	169,092 (99.8)	416 (0.2)
Takatsu (Left 1 km)	167,023	153,643 (92.0)	152,448 (91.3)	139,993 (83.8)	139,633 (99.7)	360 (0.3)
Takatsu (Right 500 m)	202,839	197,007 (97.1)	195,506 (96.4)	179,825 (88.7)	179,166 (99.6)	659 (0.4)
Takatsu (Right 1 km)	267,266	258,367 (96.7)	256,276 (95.9)	236,318 (88.4)	234,913 (99.4)	1,405 (0.6)
Sendai (High tide)	83,224	81,469 (97.9)	80,895 (97.2)	73,927 (88.8)	73,642 (99.6)	285 (0.4)
Sendai (Low tide)	170,223	166,437 (97.8)	164,967 (96.9)	155,201 (91.2)	155,148 (100)	53 (0.0)
Sendai (Left 500 m)	147,759	144,440 (97.8)	143,119 (96.9)	132,680 (89.8)	132,276 (99.7)	404 (0.3)
Sendai (Left 1 km)	177,691	171,791 (96.7)	169,645 (95.5)	157,985 (88.9)	157,856 (99.9)	129 (0.1)
Sendai (Right 500 m)	151,445	148,697 (98.2)	147,965 (97.7)	140,021 (92.5)	139,811 (99.9)	210 (0.1)
Sendai (Right 1 km)	192,176	186,251 (96.9)	184,471 (96.0)	171,052 (89.0)	170,736 (99.8)	316 (0.2)
Total	4,442,577	4,074,921 (91.7)	4,040,287 (90.9)	3,745,017 (84.3)	3,735,583 (99.7)	9,434 (0.3)
Aka (Negative control)	450	395 (87.8)	387 (86.0)	287 (63.8)	280 (97.6)	7 (2.4)
Tama (Negative control)	14,815	14,338 (96.8)	14,174 (95.7)	12,811 (86.5)	12,811 (100.0)	0 (0.0)
Miya (Negative control)	118	102 (86.4)	98 (83.1)	8 (6.8)	8 (100.0)	0 (0.0)
Takatsu (Negative control)	870	804 (92.4)	797 (91.6)	594 (68.3)	49 (8.2)	545 (91.8)
Sendai (Negative control)	1,020	915 (89.7)	897 (87.9)	690 (67.7)	235 (34.1)	455 (65.9)
1st PCR blank-1	271	253 (93.4)	248 (91.5)	211 (77.9)	0 0.0	211 (100)
1st PCR blank-2	22	17 (77.3)	16 (72.7)	12 (54.6)	0 0.0	12 (100)
1st PCR blank-3	0	0	0	0	0	0
1st PCR blank-4	5	4 (80.0)	4 (80.0)	0 (0.0)	0 (0.0)	0 (0.0)
1st PCR blank-5	1	1 (100)	0 (0.0)	0 (0.0)	0 (0.0)	0 (0.0)
1st PCR blank-6	2	0	0	0	0	0
1st PCR blank-7	8	7 (87.5)	7 (87.5)	0 (0.0)	0 (0.0)	0 (0.0)
1st PCR blank-8	18	17 (94.4)	17 (94.4)	6 (33.3)	0 0.0	6 (100)
2nd PCR blank-1	0	0	0	0	0	0
2nd PCR blank-2	0	0	0	0	0	0
Total	17,600	16,853 (95.8)	16,645 (94.6)	14,619 (83.1)	13,383 (91.5)	1,236 (8.5)
