# Supplementary material for: Evaluation of fish biodiversity in estuaries using environmental DNA metabarcoding
Source: PLoS One. 2020 Oct 6;15(10):e0231127. doi: 10.1371/journal.pone.0231127 (PMC7538199; doi:10.1371/journal.pone.0231127)
Supplement: S2 Table — Plus (+) represents occurrence. HT: river mouth at high tide; LT: river mouth at low tide; L1: left 500 m; L2: left 1 km; R1: right 500 m; R2: right 1 km. †: endangered species according to the IUCN (https://www.iucnredlist.org). ‡: endangered species according to the Ministry of the Environment of Japan (http://ikilog.biodic.go.jp/Rdb/env). †‡: endangered species according to both classifications. (PDF) [file pone.0231127.s003.pdf]

S2 Table.

Family	Scientific Name	Aka						Tama				Miya						Takatsu						Sendai					
		HT	LT	L1	L2	R1	R2	HT	LT	L2	R1	HT	LT	L1	L2	R1	R2	HT	LT	L1	L2	R1	R2	HT	LT	L1	L2	R1	R2
Dasyatidae	*Hemitrygon akajei* †												+	+															
Anguillidae	*Anguilla japonica* †‡											+	+	+	+			+	+	+	+	+	+	+	+	+			
Muraenidae	*Gymnothorax kidako*																											+	
Engraulidae	*Engraulis japonicus*		+	+	+	+	+	+	+	+	+		+		+							+		+	+	+	+	+	+
Clupeidae	*Etrumeus micropus*							+			+			+	+											+			
	Konosirus punctatus		+	+	+	+	+	+	+	+	+	+	+	+	+		+					+	+	+	+	+	+	+	+
	Sardinella zunasi													+															
	Sardinops melanostictus					+		+		+					+		+						+						
	Spratelloides gracilis																							+		+	+	+	+
Cyprinidae	*Acheilognathus rhombeus*													+															
	Carassius spp.	+	+									+	+	+	+		+	+	+	+	+	+	+		+	+			
	Cyprinus carpio †	+	+		+	+	+			+		+	+	+	+			+	+	+	+	+	+	+	+	+			
	Gnathopogon elongatus elongatus	+	+									+	+	+	+		+												
	Hemibarbus labeo	+	+									+	+	+															
	Nipponocypris sieboldii													+															
	Nipponocypris temminckii											+						+	+	+	+	+	+	+					
	Opsariichthys platypus	+	+									+	+	+	+			+	+	+	+	+	+		+				
	Phoxinus lagowskii steindachneri	+	+																										
	Phoxinus oxycephalus jouyi		+															+	+			+	+						
	Phoxinus sp.				+	+																							
	Pseudogobio esocinus esocinus	+	+									+	+	+	+			+	+	+	+	+	+						
	Pseudorasbora parva	+																											
	Pungtungia herzi																	+	+	+	+	+	+						
	Rhodeus ocellatus ocellatus		+									+	+	+			+												
	Sarcocheilichthys variegatus ‡	+	+											+															
	Squalidus gracilis																	+	+	+		+	+						
	Squalidus japonicus														+														
	Tanakia lanceolata ‡											+	+	+	+														
	Tanakia limbata											+	+	+			+	+	+	+			+						
	Tribolodon brandtii maruta	+						+	+																				
	Tribolodon hakonensis	+	+	+	+	+	+		+			+	+	+	+		+	+	+	+	+	+	+						
Cobitidae	*Cobitis minamorii tokaiensis*											+		+															
	Cobitis takatsuensis †‡																	+	+	+		+	+						
	Cobitis sp. BIWAE type B											+			+														
	Misgurnus anguillicaudatus ‡	+	+	+		+	+							+					+	+		+	+						
	Misgurnus sp. Clade A		+																										
Balitoridae	*Barbatula* sp.																+												
	Lefua echigonia		+																										
Siluridae	*Silurus asotus*	+	+											+				+	+	+	+	+	+						
Plotosidae	*Plotosus japonicus*																				+								+
Bagridae	*Tachysurus nudiceps*																	+	+	+	+	+	+						
Osmeridae	*Plecoglossus altivelis altivelis*											+	+	+				+	+	+	+	+	+						
	Salangichthys ishikawae													+															
Salmonidae	*Oncorhynchus masou* ‡	+	+															+	+	+	+	+	+						
	Oncorhynchus mykiss	+																											
Mugilidae	*Liza affinis*																							+	+	+			
	Liza haematocheila	+	+																+										
	Mugil cephalus	+	+			+	+	+	+		+	+	+	+	+	+	+	+	+	+	+	+	+	+	+	+	+	+	+
Atherinidae	*Atherion elymus*																											+	+
	Hypoatherina tsurugae																											+	
	Hypoatherina valenciennei													+	+														
Exocoetidae	*Cypselurus* spp.			+	+	+	+													+	+								
Hemiramphidae	*Hyporhamphus sajori*			+	+							+	+	+	+		+			+	+	+	+						
Belonidae	*Ablennes hians*	+	+																	+	+							+	
	Strongylura anastomella	+	+	+	+	+	+									+	+			+	+	+	+	+	+	+			
	Tylosurus crocodilus																							+	+	+	+		
Syngnathidae	*Hippocampus mohnikei* †													+															
Scorpaenidae	*Sebastes* spp.			+									+		+														
	Sebastiscus marmoratus														+					+						+		+	+
Platycephalidae	*Platycephalus* sp. 2						+		+					+	+	+	+			+		+	+			+			
Hexagrammidae	*Hexagrammos* spp.			+		+														+	+								
Cottidae	*Cottus kazika* ‡	+										+				+		+	+	+	+	+	+						
	Cottus pollux ‡	+	+			+												+	+	+	+	+	+						
	Cottus reinii ‡											+	+	+															
Moronidae	*Lateolabrax japonicus*	+	+	+	+	+	+	+	+	+	+	+	+	+	+	+	+	+	+	+	+	+	+	+	+	+	+	+	

S2 Table. Continued.

Family	Scientific Name	Aka						Tama				Miya						Takatsu						Sendai					
		HT	LT	L1	L2	R1	R2	HT	LT	L2	R1	HT	LT	L1	L2	R1	R2	HT	LT	L1	L2	R1	R2	HT	LT	L1	L2	R1	R2
Moronidae	*Lateolabrax latus*											+	+	+	+	+	+			+	+	+	+	+	+	+	+	+	+
	Lateolabrax maculatus																								+	+			
Serranidae	*Diploprion bifasciatum*																			+									
	Epinephelus akaara †																			+			+						
	Epinephelus awoara																							+					
Centrarchidae	*Micropterus salmoides*																	+											
Apogonidae	*Apogon coccineus*																											+	
	Apogon unicolor																							+					
	Ostorhinchus doederleini																											+	+
	Ostorhinchus notatus																											+	+
	Ostorhinchus semilineatus																				+							+	+
Sillaginidae	*Sillago japonica*	+	+	+	+	+	+							+	+		+	+		+	+	+	+	+	+	+	+	+	+
Carangidae	*Caranx ignobilis*																							+					
	Decapterus maruadsi																			+									
	Kaiwarinus equula																				+								
	Seriola dumerili																							+					
	Seriola quinqueradiata	+																		+									
	Trachurus japonicus †	+		+	+	+	+													+	+	+	+	+	+	+	+	+	+
Leiognathidae	*Equulites rivulatus*																									+		+	
	Nuchequula nuchalis	+	+	+		+	+								+														
Lutjanidae	*Lutjanus ophuysenii*																				+								
Gerreidae	*Gerres equulus*																												+
Haemulidae	*Parapristipoma trilineatum*													+						+		+					+		
	Plectorhinchus cinctus																							+	+	+	+		
Sparidae	*Acanthopagrus latus*									+	+	+												+	+	+	+		
	Acanthopagrus schlegelii	+		+	+	+	+	+	+	+	+	+	+	+	+	+	+	+	+	+	+	+	+	+	+	+	+	+	+
	Pagrus major		+		+		+															+		+		+	+	+	+
	Rhabdosargus sarba												+		+													+	
Polynemidae	*Polydactylus plebeius*																										+		
Sciaenidae	*Nibea mitsukurii*																							+		+			
	Pennahia argentata				+	+					+																		
Mullidae	*Parupeneus ciliatus*																										+		
Pempheridae	*Pempheris* sp.																											+	+
Kyphosidae	*Girella leonina*																									+	+	+	+
	Girella punctata	+		+		+		+			+	+					+			+	+	+		+		+	+	+	+
	Kyphosus bigibbus																												+
	Microcanthus strigatus																										+		
Chaetodontidae	*Chaetodon auripes*																											+	+
Teraponidae	*Rhynchopelates oxyrhynchus*																				+	+	+	+	+	+	+		+
Oplegnathidae	*Oplegnathus fasciatus*																				+								
Cheilodactylidae	*Goniistius* sp.																				+								
Embiotocidae	*Ditrema temminckii*										+												+						
Pomacentridae	*Abudefduf* spp.																											+	+
	Pomacentrus coelestis																												+
	Pomacentrus nagasakiensis																												+
Labridae	*Choerodon azurio*																				+								
	Halichoeres poecilopterus																			+	+								
	Halichoeres tenuispinis																										+		+
	Pseudolabrus eoethinus																											+	+
	Pseudolabrus sieboldi																												+
	Stethojulis interrupta																											+	
	Thalassoma lunare																												+
	Thalassoma cupido																											+	+
Stichaeidae	*Dictyosoma burgeri* form b																			+		+				+			
Pholidae	*Pholis nebulosa*														+									+					
Tripterygiidae	*Enneapterygius etheostomus*			+																+	+							+	
	Enneapterygius sp.																												+
Blenniidae	*Entomacrodus stellifer*																			+	+			+		+	+		+
	Istiblennius enosimae																										+	+	+
	Omobranchus elegans			+										+		+					+								
	Omobranchus fasciolatoceps																+							+		+			
	Omobranchus loxozonus																												+
	Omobranchus punctatus										+					+	+					+	+	+	+				
	Parablennius yatabei			+		+		+							+					+	+	+	+	+		+	+	+	+
	Petroscirtes breviceps																												+
Callionymidae	*Repomucenus valenciennei*										+																		
	Repomucenus spp.				+																								

S2 Table. Continued.

Family	Scientific Name	Aka						Tama				Miya						Takatsu						Sendai					
		HT	LT	L1	L2	R1	R2	HT	LT	L2	R1	HT	LT	L1	L2	R1	R2	HT	LT	L1	L2	R1	R2	HT	LT	L1	L2	R1	R2
Odontobutidae	*Odontobutis hikimius* ‡																		+										
	Odontobutis obscura																	+	+	+	+	+	+						
Eleotridae	*Eleotris oxycephala*																	+	+	+	+	+	+						
Gobiidae	*Acanthogobius flavimanus*	+	+						+	+	+	+	+	+	+	+	+			+			+		+	+			
	Acanthogobius lactipes												+									+	+						
	Acentrogobius spp.																							+					
	Bathygobius hongkongensis																									+			
	Chaenogobius annularis			+			+																						
	Chaenogobius gulosus			+																									
	Eutaeniichthys gilli ‡																									+	+		
	Favonigobius gymnauchen															+													
	Glossogobius olivaceus																							+					
	Gymnogobius breunigii								+			+	+	+	+	+	+												
	Gymnogobius castaneus ‡	+	+			+																							
	Gymnogobius heptacanthus													+															
	Gymnogobius opperiens		+																										
	Gymnogobius petschiliensis	+												+				+	+	+	+	+	+						
	Gymnogobius scrobiculatus ‡											+	+	+									+						
	Gymnogobius urotaenia	+	+	+										+	+		+		+	+		+							
	Istigobius campbelli																												+
	Luciogobius guttatus	+		+								+	+	+		+	+	+	+	+		+	+						
	Luciogobius pallidus																	+	+	+		+	+						
	Luciogobius platycephalus													+	+	+	+												
	Redigobius bikolanus																			+	+								
	Rhinogobius similis											+	+	+	+			+	+	+	+	+	+			+	+		
	Rhinogobius spp.	+	+									+		+	+			+	+	+	+	+	+	+	+		+		
	Taenioides snyderi																								+	+	+		
	Tridentiger trigonocephalus								+															+		+	+		
	Tridentiger spp.	+	+									+	+	+		+	+	+	+	+	+	+	+	+	+	+	+		
Ptereleotridae	*Parioglossus dotui*																								+				
Scatophagidae	*Scatophagus argus*																							+	+	+	+	+	
Siganidae	*Siganus fuscescens*													+	+					+	+	+	+			+		+	
Acanthuridae	*Prionurus scalprum*																							+					
Sphyraenidae	*Sphyraena japonica*																							+		+			
	Sphyraena pinguis				+			+												+	+	+	+	+		+	+	+	+
	Sphyraena obtusata																											+	
Scombridae	*Auxis* spp.																							+					
	Scomber spp.			+	+	+		+		+										+			+						
	Scomberomorus niphonius																						+						
Paralichthyidae	*Paralichthys olivaceus*	+		+	+	+	+							+	+		+												
Pleuronectidae	*Kareius bicoloratus*								+			+	+	+	+	+	+			+									
	Platichthys stellatus					+																							
	Pseudopleuronectes yokohamae													+	+														
Soleidae	*Heteromycteris japonicus*				+																								
Cynoglossidae	*Paraplagusia japonica*																+												
Monacanthidae	*Rudarius ercodes*						+																						
	Stephanolepis cirrhifer																			+	+							+	+
Ostraciidae	*Ostracion* spp.																												+
Tetraodontidae	*Canthigaster rivulata*																											+	+
	Takifugu spp.	+	+	+	+	+	+			+		+	+	+	+	+	+	+		+	+	+	+	+	+	+	+	+	+
Number of Species		37	34	25	20	25	19	12	12	10	13	34	32	51	39	17	29	32	33	59	50	49	51	41	27	43	33	39	42
Station		HT	LT	L1	L2	R1	R2	HT	LT	L2	R1	HT	LT	L1	L2	R1	R2	HT	LT	L1	L2	R1	R2	HT	LT	L1	L2	R1	R2
River		Aka						Tama				Miya						Takatsu						Sendai					
Number of Species (Total)		64						25				72						81						94					
Number of Endangered Species (IUCN)†		2						1				4						5						3					
Number of Endangered Species (Japan)‡		6						0				7						8						2					
